# Atlanta Medical College

**Published:** 1856-07

**Authors:** 


### EDITORIAL AND MISCELLANEOUS.

### ATLANTA MEDICAL COLLEGE.

We stated in our last issue that the number of matriculates in the above Institution, at that date, was larger than that of the previous session at its close. We are happy to say that, since that issue, there has been a number of additions to the class; there being at present, the rise of 100 matriculates, and from all we can learn, we think the number will still be increased, and will, perhaps, reach from 110 to 115 before the close of the term. This is certainly a handsome and healthy increase over last session, and is, undoubtedly, indicative of the prosperity of the Institution. But, if there still be some who doubt the permanency of the Institution, (and we hear of none, save those in *high* places, and who would rejoice over its downfall,) we would ask them to point us to a Medical College with a greater proportionate increase. This, perhaps, to some, may savor too much of boasting, and we would likely think so ourselves, did we not know that the impression has, in more than one instance, been attempted to be made upon students proposing to attend a course of lectures in Atlanta, that the Institution would be short lived, &c. But we will not stop here to discuss this feature of the subject, as the day has long since passed when the friends of the School, even the most lukewarm, entertained the least doubt in regard to its permanent prosperity; and were we disposed to enter into such a discussion, it is evident, that however forcible the data or the arguments deduced, they would fall far short of convincing those who allow their prejudices to pervert their better judgment.

The friends of the Institution are extremely gratified at its success; but none feel so much as those upon whom its management has devolved—I mean the Faculty—those who, in its darkest hour, stood unmoved; resisting with their every energy, the storm which threatened to overwhelm them; receiving alike, with meekness, the encouragement of friends, and the abuse of enemies. Conscious of the greatness of the enterprise, and the correctness of the principles which directed them, they were content, for the time, to submit to all that might be said derogatory to their interests, both individually and as a body, feeling that time was only necessary for them to receive justice at the hands of a discriminating public. Nor have they been deceived in this particular, as all has, long since, been awarded that the Faculty could ask.

And we are happy too, to say, that all unpleasant feelings in their midst, towards the School, has long since passed away, and that the Institution numbers now among its strongest friends those who opposed it in the outset. This is highly gratifying to the Faculty, and, we conceive, too, quite complimentary, to thus be able, where their works, whether good or bad are noted, to convince even those who opposed them of the correctness of their purpose.

It was confidently expected, at the close of the previous session, that the College edifice would be in readiness for the present course of lectures, but from the combination of circumstances mentioned in a previous number of this Journal, and entirely beyond the control of the Faculty, it was not thought practicable. We are happy, now, however, to state, that the College building, with all the recent improvements in such buildings, is going up, and will, without doubt, be in readiness for the next course of lectures. We did propose in this issue, to give a description of the College edifice, but as we hope in a few months to present an engraved view of the building as an embelishment to the cover of our Journal, we will defer it until then.

Our Museum, which, during the past session was small, and at present, when compared with some of the older Institutions is not very large, is gradually increasing both by purchase and the contribution of friends. And in this connection we cannot forbear mentioning some beautiful wax preparations recently presented by Dr. N. D'Alvigny, preparations made by himself, and illustrating some important abnormal deviations.

Although we have all that is essential to demonstrate a course of lectures, still our pathological, surgical and anatomical cabinets are not yet as extensive as we would desire them.

One or two members of the Faculty will leave for Europe at the close of the present session, and during their stay will make such other additions as are thought desirable.

